# Supplementary figures and images for: Predictors of loneliness among middle childhood and adolescence during the COVID-19 pandemic
Source: PLoS One. 2024 Aug 15;19(8):e0308091. doi: 10.1371/journal.pone.0308091 (PMC11326567; doi:10.1371/journal.pone.0308091)

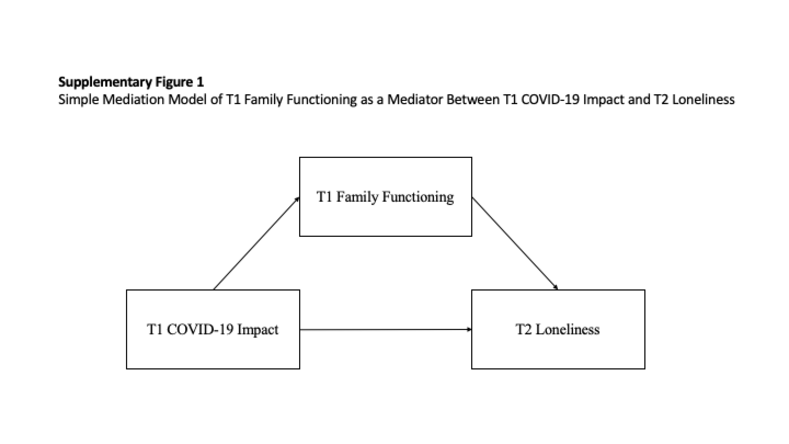

Supplement: S1 Fig — (TIF) [file pone.0308091.s001.tif]

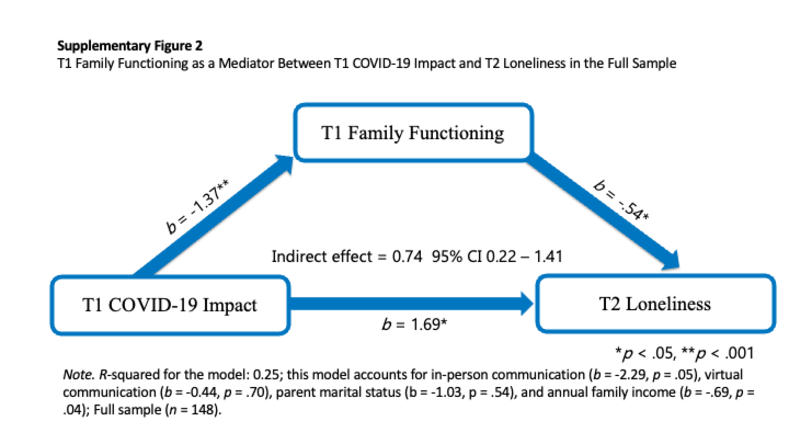

Supplement: S2 Fig — (TIF) [file pone.0308091.s002.tif]

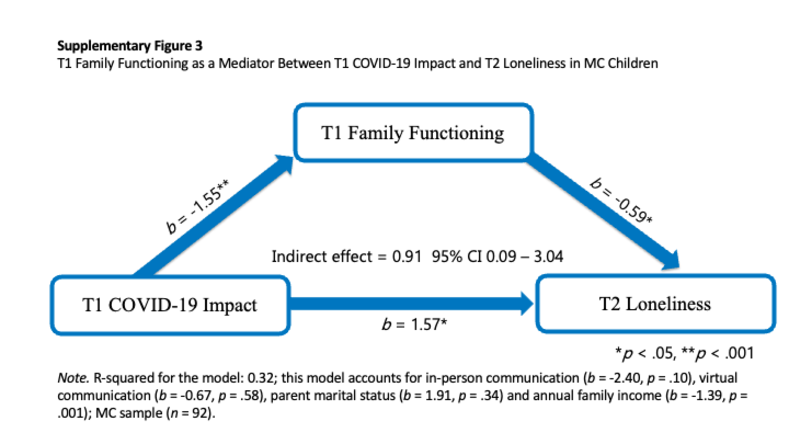

Supplement: S3 Fig — (TIF) [file pone.0308091.s003.tif]
