# Supplementary material for: Predictors of loneliness among middle childhood and adolescence during the COVID-19 pandemic
Source: PLoS One. 2024 Aug 15;19(8):e0308091. doi: 10.1371/journal.pone.0308091 (PMC11326567; doi:10.1371/journal.pone.0308091)
Supplement: S1 Table — (DOCX) [file pone.0308091.s004.docx]

**Supplementary Table 1**

*Comparisons on Variables of Interest for MC Children and Adolescents Present at only T1 versus both T1 and T2*

|  | Present at only T1 | | Present at both T1 and T2 | | *t-value* | *p* | Cohen's *d* |
| --- | --- | --- | --- | --- | --- | --- | --- |
|  | *M* | *SD* | *M* | *SD* |  |  |  |
| **MC Children**  Child age | 10.03 | 1.33 | 10.03 | 1.48 | -0.03 | .98 | 1.38 |
| T1 Family functioning | 48.22 | 7.93 | 48.90 | 8.00 | -0.67 | .50 | 7.95 |
| T1 COVID impact | 2.97 | 0.87 | 2.93 | 0.84 | 0.36 | .72 | 0.86 |
| Prior family income | 4.04 | 1.56 | 4.35 | 1.46 | -1.56 | .12 | 1.52 |
| T1 Loneliness | 55.24 | 10.64 | 54.30 | 11.11 | 0.68 | .50 | 10.80 |
| **Adolescents** |  |  |  |  |  |  |  |
| Child age | 14.80 | 1.42 | 14.66 | 1.41 | 0.62 | .54 | 1.41 |
| T1 Family functioning | 45.65 | 7.59 | 46.94 | 7.72 | -1.05 | .29 | 7.63 |
| T1 COVID impact | 3.08 | 0.84 | 3.07 | 0.82 | 0.09 | .93 | 0.84 |
| Prior family income | 4.16 | 1.52 | 4.11 | 1.67 | 0.22 | .83 | 1.57 |
| T1 Loneliness | 57.80 | 12.72 | 58.64 | 9.77 | -0.44 | .66 | 11.87 |

*Note*. Prior family income was measured on a 1 to 6 scale with the scores being: 1 for “Under $25,000”, 2 for “$25,001-$50,000”, 3 for “50,001-$75,000”, 4 for “75,001-$100,000”, 5 for “100,001-$150,000”, and 6 for “150,000-more.” There was also a 7 option for “Other.”
