# Supplementary material for: Predictors of loneliness among middle childhood and adolescence during the COVID-19 pandemic
Source: PLoS One. 2024 Aug 15;19(8):e0308091. doi: 10.1371/journal.pone.0308091 (PMC11326567; doi:10.1371/journal.pone.0308091)
Supplement: S2 Table — (DOCX) [file pone.0308091.s005.docx]

| **Supplementary Table 2**  *Examining Differences in MC Children Present at just T1 and both T1 and T2 through Chi-Square Tests* | |  |  |  |
| --- | --- | --- | --- | --- |
| Independent variables | Present just T1 (%) | Present both T1 T2 (%) | χ^2^value | p-value |
| Child sex |  |  |  |  |
| Male | 48.9 | 57.6 |  |  |
| Female | 51.1 | 42.4 | 1.86 | .17 |
| Child race |  |  |  |  |
| White | 89.1 | 93.5 |  |  |
| Not White | 10.9 | 6.5 | 1.40 | .24 |
| Child ethnicity |  |  |  |  |
| Hispanic | 8.7 | 8.7 |  |  |
| Non-Hispanic | 91.3 | 91.3 | 0.00 | 1.00 |
| Communication with friends |  |  |  |  |
| In-person | 19.6 | 18.5 |  |  |
| Not in-person | 80.4 | 81.5 | 0.05 | .83 |
| Virtual | 76.1 | 69.6 |  |  |
| Not virtual | 23.9 | 30.4 | 1.35 | .25 |
| Parent employment status |  |  |  |  |
| Employed | 76.1 | 67.4 |  |  |
| Unemployed | 23.9 | 32.6 | 2.36 | .12 |
| Parent marital status |  |  |  |  |
| Married or has a partner | 85.8 | 91.3 |  |  |
| Not married or no partner | 14.2 | 8.7 | 0.19 | .25 |
