# Supplementary material for: Predictors of loneliness among middle childhood and adolescence during the COVID-19 pandemic
Source: PLoS One. 2024 Aug 15;19(8):e0308091. doi: 10.1371/journal.pone.0308091 (PMC11326567; doi:10.1371/journal.pone.0308091)
Supplement: S3 Table — (DOCX) [file pone.0308091.s006.docx]

| **Supplementary Table 3**  *Examining Differences in Adolescents Present at just T1 and both T1 and T2 through Chi-Square Tests* | |  |  |  |
| --- | --- | --- | --- | --- |
| Independent variables | Present just T1 (%) | Present both T1 T2 (%) | χ^2^value | p-value |
| Adolescent sex |  |  |  |  |
| Male | 51.3 | 48.2 |  |  |
| Female | 48.7 | 51.8 | 0.14 | .71 |
| Adolescent race |  |  |  |  |
| White | 90.1 | 92.9 |  |  |
| Not White | 9.9 | 7.1 | 0.36 | .55 |
| Adolescent ethnicity |  |  |  |  |
| Hispanic | 10.0 | 10.9 |  |  |
| Non-Hispanic | 90.0 | 89.1 | 0.03 | .85 |
| Communication with friends |  |  |  |  |
| In-person | 32.2 | 26.8 |  |  |
| Not in-person | 67.8 | 73.2 | 0.54 | .46 |
| Virtual | 87.6 | 89.3 |  |  |
| Not virtual | 12.4 | 10.7 | 0.10 | .81 |
| Parent marital status |  |  |  |  |
| Married or has a partner | 85.8 | 85.7 |  |  |
| Not married or no partner | 14.2 | 14.3 | 0.00 | .98 |
